# A peculiar case of primary lymphoma of pancreas: A rare presentation of Hodgkin lymphoma

**DOI:** 10.18632/oncoscience.628

**Published:** 2025-10-06

**Authors:** Osama Mohiuddin, Rafi Aibani, Aarish Lalani, Faiqa Shabbir, Amir Aibani, Vivek Sharma

**Affiliations:** ^1^Ascension At Agnes Healthcare, Internal Medicine Resident, WV 25304, USA; ^2^Charleston Area Medical Center, Internal Medicine Resident, WV 25304, USA; ^3^Federal Medical and Dental College, Medical Student, Pakistan; ^4^Aga Khan University, Medical Student, Pakistan; ^5^FACP, University of Louisville, Director, Medical Oncology Service, GI Cancer Multidisciplinary Clinic, Professor of Medicine, KY 40202, USA

**Keywords:** primary pancreatic lymphoma, Hodgkin lymphoma

## Abstract

Primary pancreatic Hodgkin lymphoma (PPL) is an exceptionally rare condition often misdiagnosed as pancreatic adenocarcinoma or pseudocyst. Early histopathological diagnosis is crucial for prognosis and appropriate chemotherapy.

We report a case of a 50-year-old male presenting with low-grade fever, chills, abdominal pain, and 30-pound weight loss over 10 weeks. Examination revealed significant epigastric tenderness without icterus. Laboratory findings showed leukopenia (1.2K/UL), low absolute neutrophil count (238), hyperbilirubinemia, and elevated AST/ALT (185/165), with normal lipase and amylase. Imaging revealed a 4.7 × 5.1 × 6.6 cm solid heterogeneous pancreatic head mass with multiple prominent retroperitoneal, gastro-hepatic, para-aortic, and portacaval lymph nodes. Retroperitoneal lymph node biopsy confirmed nodular sclerosis Hodgkin lymphoma with positive CD15, CD30, MUM-1, and EBV stains. Bone marrow biopsy revealed lymphoma infiltration, prompting initiation of A+AVD (brentuximab vedotin, doxorubicin, vinblastine, dacarbazine) chemotherapy for stage IV disease based on Karnofsky Performance Status.

Hodgkin lymphoma, a malignant B-cell neoplasm, constitutes 11% of lymphomas and is characterized by Reed-Sternberg cells. PPL is extremely rare, often mimicking pancreatic adenocarcinoma. Features such as absent pancreatic atrophy, tumor necrosis, calcification, and vascular invasion may aid differentiation. Treatment depends on disease stage, with early stage managed by ABVD (Adriamycin, Bleomycin sulfate, Vinblastine sulfate, Dacarbazine) chemotherapy and radiation, while advanced cases require extended A+AVD chemotherapy. PPL accounts for <2% of extra-nodal lymphomas but remains a potentially treatable entity. This case underscores the importance of obtaining tissue diagnosis in the setting of a pancreatic mass before embarking on definitive treatment.

## INTRODUCTION

Primary pancreatic lymphoma is a rare form of extra-nodal lymphoma, with diffuse large B-cell lymphoma representing the most common histologic subtype. Less frequently, primary pancreatic lymphoma may present as follicular lymphoma, small lymphocytic lymphoma, or T-cell lymphoma, and may be classified as either non-Hodgkin or Hodgkin lymphoma. Primary pancreatic Hodgkin lymphoma is exceptionally rare and is often misdiagnosed as pancreatic adenocarcinoma or pancreatic pseudocyst during early clinical evaluation [1, 2]. Prompt histopathological diagnosis is critical for timely initiation of appropriate chemotherapy and improved patient outcomes [3]. This case highlights a rare presentation of Hodgkin lymphoma involving the pancreas, aiming to raise clinical awareness, reduce diagnostic delays and avoid treatment missteps that can be associated with this unusual entity.

## CASE PRESENTATION

A Hispanic male in his early 50s presented with a 10-week history of persistent low-grade fever, intermittent chills, epigastric abdominal pain, and unintentional weight loss totaling approximately 30 pounds. On admission, his general condition was stable, and he remained functionally independent. Physical examination was notable for epigastric tenderness on deep palpation. No scleral icterus or other signs of systemic illness were observed.

Initial laboratory investigations revealed leukopenia with a white blood cell count of 1.2 K/μL and an absolute neutrophil count of 238/μL. Liver function tests demonstrated elevated transaminases (AST 185 U/L, ALT 165 U/L) and hyperbilirubinemia. Serum lipase and amylase levels were within normal limits. Blood, urine, and other infectious cultures returned negative.

**Figure 1:**
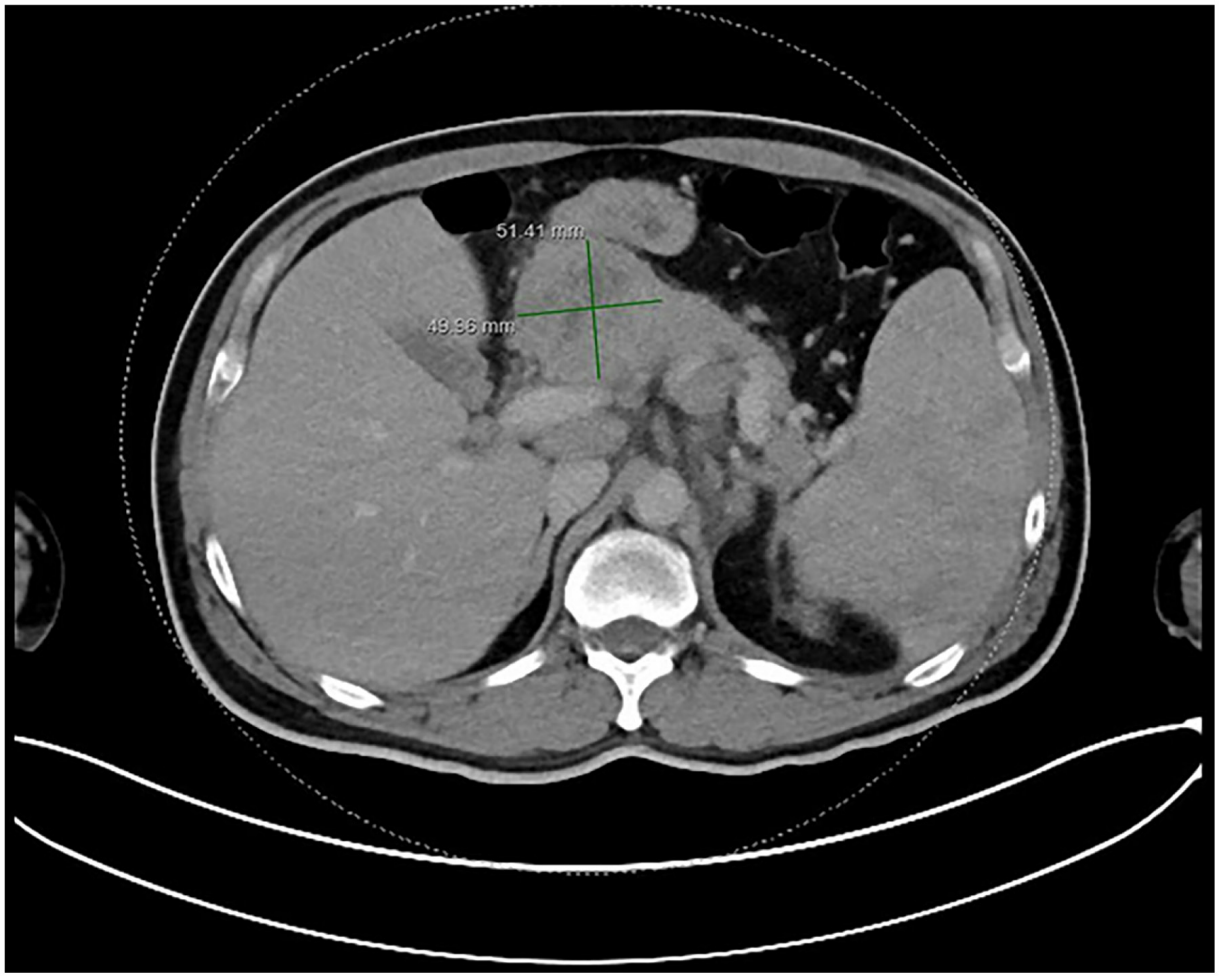
CT scan Abdomen axial view showing lesion within pancreas/adjacent peripancreatic lymphadenopathy.

An abdominal ultrasound identified a lesion in the head of the pancreas. Further imaging with a contrast-enhanced computed tomography (CT) scan of the chest, abdomen, and pelvis confirmed a solid, heterogeneous mass in the pancreatic head, measuring 4.7 × 5.1 × 6.6 cm. Multiple prominent lymph nodes were observed in the retroperitoneal, gastrohepatic, para-aortic, and portocaval regions (Figure 1). Based on imaging and clinical presentation, pancreatic adenocarcinoma was initially suspected.

**Figure 2:**
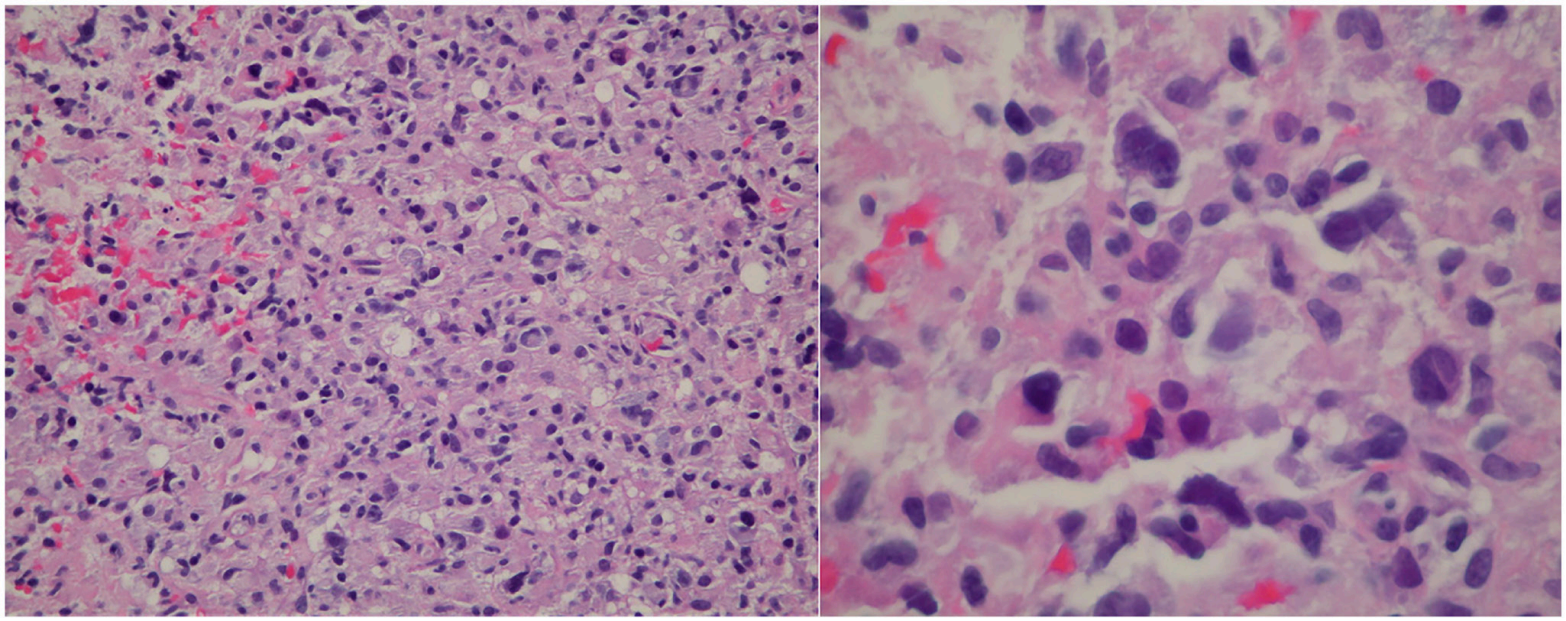
H&E stain showing, low power field left panel: Atypical lymphohistiocytic proliferation and high power field right panel showing Reed Sternberg cell variant.

**Figure 3:**
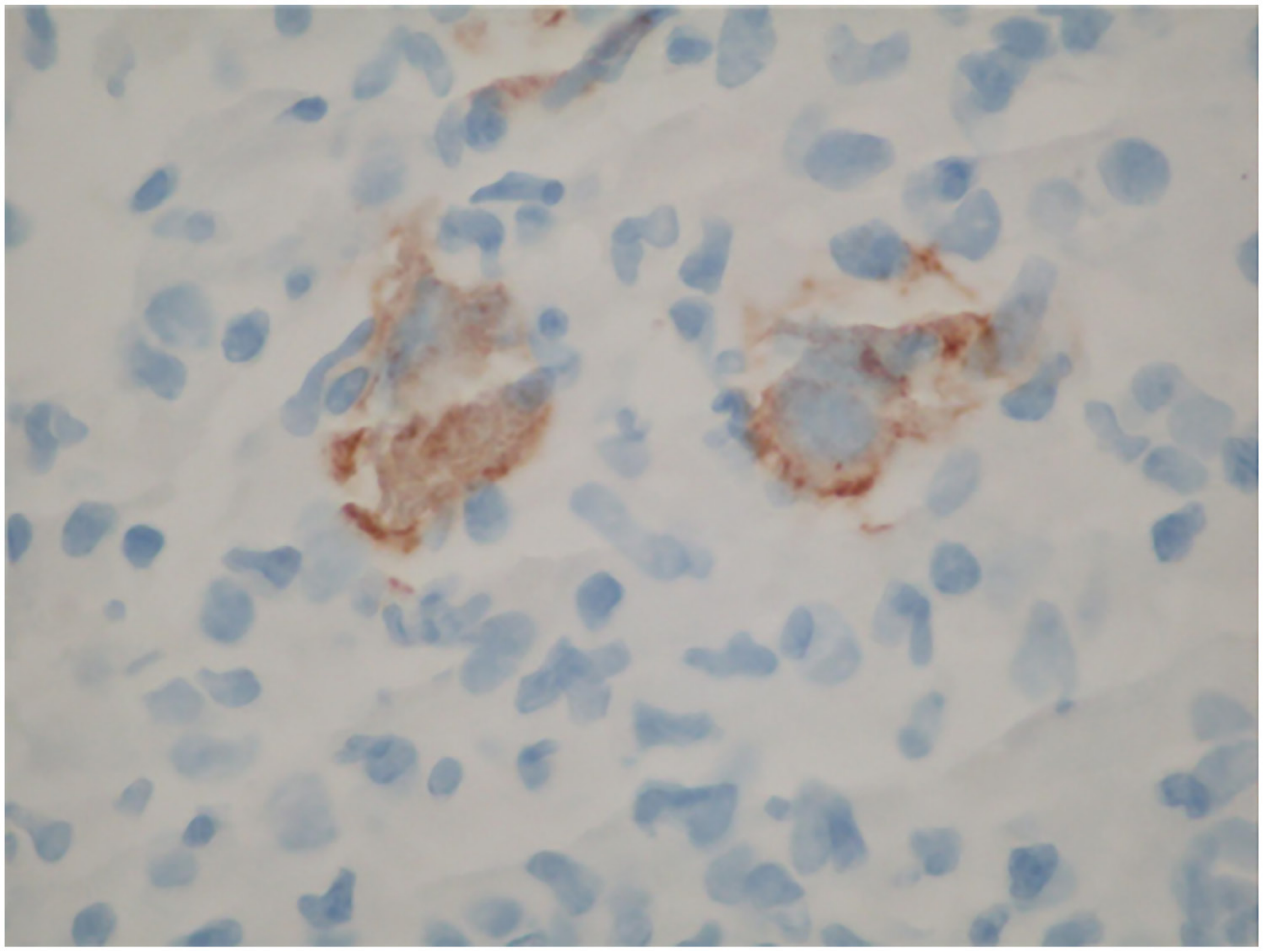
CD 30 Immunohistochemical stain showing scattered large and atypical cells that coexpress CD 15, MUM1, PAX 5 and EBV.

Given the easier accessibility of lymphadenopathy, a biopsy of a retroperitoneal lymph node was chosen over pancreatic mass sampling. Histopathological analysis revealed classic Hodgkin lymphoma of the nodular sclerosis subtype, with neoplastic cells staining positive for CD15, CD30, MUM1, and Epstein-Barr virus (EBV)-encoded RNA (EBER) (Figures 2 and 3). A subsequent bone marrow biopsy confirmed infiltration with Hodgkin lymphoma, establishing the diagnosis of stage IV disease.

Given good Karnofsky Performance Status score of 90, the patient was initiated on combination chemotherapy with the A-AVD regimen (brentuximab vedotin, doxorubicin, vinblastine, and dacarbazine). At the time of reporting, he had successfully completed the first of six planned cycles without major complications.

## DISCUSSION

Hodgkin lymphoma (HL) is a malignant B-cell neoplasm accounting for approximately 11% of all lymphomas in the United States [4]. It is broadly divided into classical Hodgkin lymphoma (cHL)—which includes the nodular sclerosis, mixed cellularity, lymphocyte-depleted, and lymphocyte-rich subtypes—and nodular lymphocyte-predominant Hodgkin lymphoma (NLPHL). Reed-Sternberg cells remain the defining histopathological hallmark. Although HL primarily involves the lymphatic system, extranodal disease occurs in ~40% of cases, most commonly affecting the spleen, liver, and gastrointestinal tract. Pancreatic involvement is exceptionally rare, with primary pancreatic Hodgkin lymphoma (PPHL) representing an unusual and sparsely reported entity [5].

Most pancreatic tumors are ductal adenocarcinomas, while only 13% fall into other histologies [1]. Primary pancreatic lymphoma (PPL) is particularly uncommon, comprising <1% of pancreatic malignancies, with nearly all cases classified as non-Hodgkin lymphoma [2]. HL of the pancreas is therefore extraordinary and diagnostically challenging due to its radiologic and clinical overlap with adenocarcinoma. Unlike adenocarcinoma, however, PPL typically lacks distal pancreatic atrophy, necrosis, calcification, or vascular invasion—features that can aid in differentiation [5]. According to WHO criteria, PPL is defined by disease bulk localized to the pancreas, with or without regional nodal or distant spread [6].

Prognosis in advanced HL is assessed using the International Prognostic Factors Project, which identifies seven adverse risk variables: age >45 years, male sex, stage IV disease, hemoglobin <10.5 g/dL, WBC >15,000/μL, lymphocyte count <600/μL, and albumin <4.0 g/dL [7]. Patients with ≥5 factors have a 5-year progression-free survival (PFS) of ~42%, compared to ~84% for those without any [7].

Management of HL is stage-dependent. Early-stage disease is generally treated with two cycles of ABVD (doxorubicin, bleomycin, vinblastine, dacarbazine) followed by involved-field radiation therapy (20 Gy) [8]. For advanced disease, systemic chemotherapy is standard, and recent trials demonstrate that A+AVD (brentuximab vedotin plus doxorubicin, vinblastine, dacarbazine) improves outcomes compared with ABVD, reducing progression or death risk and pulmonary toxicity [9].

For relapsed or refractory HL, salvage chemotherapy followed by autologous stem cell transplantation (ASCT) remains the cornerstone. In patients ineligible for ASCT, or those relapsing post-transplant, targeted therapies such as brentuximab vedotin and immune checkpoint inhibitors (anti–PD-1/PD-L1) have shown meaningful efficacy [10].

## CONCLUSIONS

Pancreatic involvement by HL remains a rare clinical entity, often mimicking pancreatic adenocarcinoma both symptomatically and radiologically, thus presenting significant diagnostic challenges. A high index of suspicion, combined with comprehensive histopathological, radiologic, and molecular assessment, is essential for accurate diagnosis. Getting tissue diagnosis of a pancreatic mass can be challenging, sometimes requiring multiple attempts at obtaining an adequate biopsy. This case highlights the value of pursuing such diagnostic accuracy. The increasing availability of targeted and immunotherapeutic agents has significantly expanded the treatment armamentarium for HL. Given the rarity of pancreatic presentations, further study is needed to enhance understanding and optimize the diagnostic and therapeutic approach to this uncommon manifestation of HL.
